# Brazilian version of the Fear of Falls Scale: translation, cross-cultural adaptation and validation

**DOI:** 10.1055/s-0045-1815738

**Published:** 2026-02-04

**Authors:** Carla Marineli Saraiva do Amaral, André Borges Ferreira Gomes, Rafael Barbosa Mokdeci Surerus, Pedro Pigozzo Senra Lacerda, Vanessa Fernandes de Oliveira, Stephanie Suzanne de Oliveira Scott, Samuel Brito de Almeida, Walberto Silva dos Santos, Thiago Cardoso Vale, Fernanda Martins Maia Carvalho, Pedro Braga-Neto

**Affiliations:** 1 Universidade Federal do Ceará, Faculdade de Medicina, Departamento de Medicina Clínica, Fortaleza CE, Brazil.; 2 Hospital Geral de Fortaleza, Serviço de Neurologia, Fortaleza CE, Brazil.; 3 Universidade Federal de Juiz de Fora, Faculdade de Medicina, Departamento de Clínica Médica, Juiz de Fora MG, Brazil.; 4 Universidade Federal do Ceará, Centro de Ciências Humanas, Departamento de Psicologia, Fortaleza CE, Brazil.

**Keywords:** Accidental Falls, Postural Balance, Parkinson disease

## Abstract

**Background:**

Fear of falling can be present in the daily lives of patients with Parkinson's disease (PD) due to their predisposition to falls.

**Objective:**

The main objective of this study was to translate the Fear of Falls Scale (FFS) into Brazilian Portuguese, adapt it cross-culturally, and validate it.

**Methods:**

A multicenter, cross-sectional study was conducted with PD patients fluent in Brazilian Portuguese, recruited from five research centers in Brazil. Descriptive analysis characterized the sample and compared the data. Cronbach's α and McDonald's ω coefficients were used to assess the internal consistency of the scale.

**Results:**

No significant differences were observed between the translated versions T1 and T2. The B1 and B2 versions did not present significant divergences in the back-translation from the original scale. The Movement Disorder Society – Unified Parkinson Disease Rating Scale (MDS-UPDRS) part III significantly correlated with motor skills (rho = 0.56, 0.43–0.67; *p* ≤ 0.001) and fear of falling (rho = 0.48, 0.34–0.60; *p* ≤ 0.001). Higher stages on the modified Hoehn & Yahr scale were associated with a greater decline in motor skills and a greater fear of falling ( *p* < 0.001). The total internal consistency of balance-related motor skills and fear of falling was considered sufficiently reliable. Cronbach's α values were 0.96, 0.91, and 0.90; while McDonald's ω values were 0.96, 0.92, and 0.91, respectively.

**Conclusion:**

The Brazilian version of the FFS proved to be valid and reliable for assessing fear of falling in people with PD.

## INTRODUCTION

Parkinson's disease (PD) is a progressive neurodegenerative disease characterized by a wide range of motor symptoms such as bradykinesia, tremor, rigidity, and postural instability, as well as nonmotor symptoms including hyposmia, depression, constipation, and sleep disturbances, among others. Postural reflex dysfunction compromises body stability as the disease progresses, increasing the risk of falls and potentially generating fear. 1 2 Both the history and fear of falling can be relevant risk factors for future occurrences. 1 3

A few scales have been used to measure predisposition to and fear of falling. Among them, the Fear of Falls Scale (FFS) evaluates the degree of concern or fear experienced by individuals with PD regarding the possibility of falling during daily activities involving motor skills related to postural balance. 4 This scale is of particular relevance, as postural instability and impaired balance are motor symptoms in patients with PD and represent determining factors for fall risk. By simultaneously addressing the subjective perception of this fear and its relationship with balance performance, the FFS provides greater understanding to support developing more targeted interventions in this population. Although published in 2019, the FFS had not been validated in Brazil.

Cross-cultural validation requires accurate translation, cultural adaptation, and investigation of psychometric properties. Exploratory Factor Analysis (EFA) is an important statistical technique for identifying the underlying structure of a set of observed variables, grouping them into latent factors that explain the correlations among them. This analysis enables assessing the instrument's internal consistency. The primary objective of this study was to translate, cross-culturally adapt, and validate the FFS for Brazilian clinical practice. Secondary objectives were to verify the association between the FFS and the subitems of parts I, II, III, and IV of the Movement Disorder Society – Unified Parkinson Disease Rating (MDS-UPDRS) 5 and modified Hoehn & Yahr (HY) scales, 6 as well as to conduct an EFA to examine the scale's structure in the Brazilian context.

## METHODS

A cross-sectional and multicenter study was conducted to translate, cross-culturally adapt, and validate the FFS into Brazilian Portuguese. Consent was obtained from the corresponding author of the original scale who granted permission through an email response.

The FFS is a self-assessment scale designed to assess fear of falling in PD patients. It comprises 24 items scored from 0 to 3, with higher scores indicating greater severity based on symptoms over 4 weeks, divided into four sections. Section 1 (FFS-1) has 10 questions on motor abilities related to balance; section 2 (FFS-2) has 10 questions about fear of falling. Section 3 (FFS-3) contains a descriptive question about any additional motor activity the patient avoids due to this fear. Section 4 (FFS-4) includes three questions addressing fear of falling, dyskinesia, gait freezing, and dopaminergic medication. 4 The reliability and validity of the FFS-Brazilian version were calculated by sections 1 and 2 and a cut-off score ≥ 3 to classify fear of falling. Although the main purpose is to assess this fear, FFS-1 provides context on motor skills related to balance. 4

### Participants

The study's inclusion criteria comprised PD patients diagnosed according to the MDS-UPDRS and fluent in Brazilian Portuguese. Exclusion criteria included a history of brain injuries, use of neuroleptic medication, dementia based on the Diagnostic and Statistical Manual of Mental Disorders, Fifth Edition (DSM-5), 7 and Mini-Mental State Examination (MMSE) scores below the cut-off points established for their educational level. 8

A total of 387 patients were recruited from five Brazilian research centers, 2 located in Fortaleza (state of Ceará) and 3 in Juiz de Fora (state of Minas Gerais). After applying the eligibility criteria, the final sample consisted of 140 patients: 70 participants from Hospital Universitário Walter Cantídio, Universidade Federal de Ceará and Hospital Geral de Fortaleza; and 70 from Hospital Universitário da Universidade Federal de Juiz de Fora, Hospital e Maternidade Therezinha de Jesus, and Hospital Monte Sinai.

The patients were included by convenience at each of the research centers and were informed about the study objectives. The study period was determined as the 70 patients from each region were included, and the evaluators at each center remained the same during this time. A neurologist was responsible for patient monitoring and supervision in each region.

### *Participant evaluation*

Health professionals conducted the MDS-UPDRS, 5 the modified HY scale, 6 and the MMSE. 8 The FFS-Brazilian version was used in both the pretest and validation. Evaluations took place during the maximum benefit of the medication (ON state). However, the specific levodopa dosages were not reported.

### Procedures

The method described by Beaton et al. 9 was used for translation, cross-cultural adaptation, and validation process of the FFS into Brazilian Portuguese, according to stages described below.

#### *Initial translation*

The first stage consisted of the initial translation of the FFS from English into Brazilian Portuguese by two bilingual Brazilian translators fluent in English. One was a healthcare provider aware of the study's objective, and the other was from the education field with no knowledge of the objective. Two versions were created (T1) and (T2) from the original scale's translations.

#### *Synthesis of the translations*

At this stage, the two versions (T1 and T2) were condensed into a single final synthesized version (T12) by consensus between a mediator and the two translators. The mediator had not participated in the previous stage and was impartial.

#### *Back-translation*

Two native English translators who did not work in healthcare generated two new versions, B1 and B2, in English, which were back-translated from T12. The translators were unaware of the original version and the study's objective. The B1 and B2 versions were created to verify validity and point out inconsistencies and conceptual errors in the translation processes.

#### *Expert committee*

An expert committee of five professionals with expertise in healthcare and scale validation evaluated the cross-cultural equivalence of the translations and the consistency of back-translations. Semantic, idiomatic, experiential, and conceptual equivalences were reviewed. Discrepancies were resolved by consensus through meetings and voting, with validity set at ≥ 80% agreement. 10 A final version was then established after all decisions and revisions were incorporated.

#### *Pretest*

In this phase, a pretest was applied to patients with version T12. The sample size (n = 30) followed the method proposed by Beaton et al. 9 The objective of the pretest was to evaluate understanding of the FFS-Brazilian version. Then, a new analysis and adaptation of words and phrases was performed, creating the final version for validation.

#### *Evaluation of the adaptation process*

In this step, all FFS - Brazilian version reports and forms were sent to the committee of experts to verify the adaptation process.

#### *FFS validation*

After completing the pretest, the process of validating the FFS for Brazilian Portuguese was undertaken. The patients were interviewed during routine consultations with their neurologist. Data collection occurred between June 2023 and March 2024.

### Ethical aspects

This study was approved by the Ethics Committees of the participating centers on CAAE: 66681823.5.1001.5045. All patients gave written informed consent to participate in this study.

### Statistical analysis

Data were analyzed using the REDCap platform (Vanderbilt University) and jamovi (The jamovi project) software, version 1.8. Descriptive statistics were used for group comparisons, and continuous variables were analyzed with the Mann-Whitney U test. Internal consistency was assessed using Cronbach's alpha (α) and McDonald's omega (ω), with acceptable reliability defined as values above 0.70. 11 12 Correlations, including strength and direction, were evaluated using Spearman's test. The *p* -values were calculated using the Mann-Whitney U test, Chi-squared test, Fisher's exact test, and Kruskal-Wallis test.

Psychometric analyses were additionally performed to investigate the internal structure of the FFS-Brazilian version. Data suitability was verified using the Kaiser–Meyer–Olkin (KMO) index (acceptable if ≥ 0.80) and Bartlett's test of sphericity ( *p* < 0.05). An EFA was conducted using polychoric correlation matrices, the Robust Diagonally Weighted Least Squares (RDWLS) via the FACTOR (Universitat Rovira i Virgili) software. 13 The number of factors to be retained was determined using both the Kaiser criterion (eigenvalue ≥ 1.0) and the optimal implementation of parallel analysis. 14

Following EFA, confirmatory factor analyses (CFA) were conducted to compare the fit of unidimensional and bifactor models. Model fit was evaluated using Root Mean Square Error of Approximation (RMSEA) with < 0.08 being considered acceptable and < 0.05 good, Comparative Fit Index (CFI ≥ 0.95), Tucker–Lewis's Non-Normed Fit Index (NNFI ≥ 0.95), Goodness-of-Fit Index (GFI ≥ 0.90), and the Chi-squared/degrees of freedom ratio (χ ^2^ /df < 2.0). The Weighted Root Mean Square Residual (WRMR < 1.0) was also reported for ordinal data. 15

## RESULTS

### Summary of translations

Overall, there were no significant differences comparing the translated versions T1 and T2. Small variations in question-and-answer formulations were detected. The T1 and T2 records and the synthesized T12 version are detailed in **Supplementary Material 1** (available at https://www.arquivosdeneuropsiquiatria.org/wp-content/uploads/2025/10/ANP-2025.0269-Supplementary-Material-1.docx ).

### Back-translation

The B1 and B2 versions were made to ensure that T12 preserved the same content as the original version. After analysis, B1 and B2 did not present major divergences compared to the original scale ( **Supplementary Material 2** , available at https://www.arquivosdeneuropsiquiatria.org/wp-content/uploads/2025/10/ANP-2025.0269-Supplementary-Material-2.docx ).

### Pretest

Pretest data collection took place from March to May 2023 with a convenience sample. During the scale application, minor adjustments were made for clarity. In question 21, *describe* was replaced by *write* . In question 22, *when the effect of the medication was wearing off* was replaced with *when the medicine lost its effect* . In question 23, *dyskinesia* was better detailed, and, in question 24, the initial doubt about *freezing* was resolved without altering the item.

### Validation and correlations

#### *Demographic and clinical characteristics of patients with and without fear of falling*

Among patients with fear of falling (n = 89), 55% were from Juiz de Fora and 45%, from Fortaleza. The mean, standard deviation (SD), and median scores for patients with and without fear of falling were as follows: 10.5 ± 5.3 (10.0) and 2.8 ± 2.5 (2.0) in FFS-1; 11 ± 5 (10) and 1 ± 1 (1) in FFS-2; and 21 ± 10 (19) and 4 ± 4 (3) in total FFS, respectively. Demographic and clinical characteristics are detailed in Table 1 .

**Table 1:** Demographic and clinical characteristics of PD patients with and without fear of falling

	PD patients with fear of falling (n = 89)	PD patients without fear of falling (n = 51)	*p* -value ^c^
Mean age in years (median)	65 ± 13 (66)	64 ± 10 (65)	0.790
Male gender: n (%)	50 (56)	38 (75)	0.031
Mean disease duration in years (median)	9.4 ± 6.1 (9.0)	6.6 ± 4.5 (5.0)	0.003
Mean MMSE score (median)	26.90 ± 1.98 (27.00)	27.57 ± 2.32 (28.00)	0.014
Mean MDS-UPDRS score (median)			
Part I	12 ± 7 (11)	8 ± 5 (7)	< 0.001
Part II	18 ± 8 (16)	8 ± 6 (7)	< 0.001
Part III	37 ± 16 (35)	23 ± 11 (19)	< 0.001
Part IV	4.8 ± 3.7 (4.0)	1.6 ± 2.2 (0.0)	< 0.001
Stage on the Modified Hoehn & Yahr scale: n (%)			< 0.001
0	0 (0)	0 (0)	
1	1 (1.1)	7 (14)	
1.5	1 (1.1)	1 (2)	
2	38 (43)	39 (76)	
2.5	9 (10)	0 (0)	
3	25 (28)	4 (7.8)	
4	15 (17)	0 (0)	
5	0 (0)	0 (0)	

Abbreviations: MDS-UPDRS, Movement Disorders Society – Unified Parkinsons' Disease Rating Scale; MMSE, Mini-Mental State Examination; PD, Parkinson's Disease.

Notes: ^a^ Mann-Whitney U test; Chi-square test of independence; Fisher's exact test.

#### *Frequency of FFS-1 and FFS-2 responses*

The frequency of responses for the FFS-1 was as follows: 44.7% for a score of 0 (no difficulty); 37.0% for 1 (little difficulty); 14.6% for 2 (very difficult); and 3.5% for 3 (unable to perform). And the frequency in relation to the FFS-2 was as follows: 51.3% for a score of 0 (no fear); 27.5% for 1 (little fear); 17.6% for 2 (very afraid); and 3.5% for 3 (unable to perform).

#### *MDS-UPDRS part III and FFS-1 versus FFS-2 correlations*

Spearman's correlation coefficient between MDS-UPDRS part III and FFS-1 (rho = 0.56; 0.43–0.67; *p* ≤ 0.001) and MDS-UPDRS III and FFS-2 (rho = 0.48; 0.34–0.60; *p* ≤ 0.001) were statistically significant with a positive correlation between FFS and MDS-UPDRS III scores.

#### *Modified HY scale and FFS-1 versus FFS-2 correlations*

The HY stage was organized into three groups according to the scale's original validation study 11 to ensure a minimum number of patients and perform correlations with the FFS-1 and FFS-2 scores. The results were statistically significant in all groups, with positive correlation between FFS and modified HY scale, as shown in Table 2 .

**Table 2:** Modified Hoehn & Yahr stages and FFS-1 versus FFS-2 scores

Characteristics	Group 1 (n = 87)	Group 2 (n = 38)	Group 3 (n = 15)	*p* -value ^a^
Mean FFS-1 score (median)	5.2 ± 4.6 (5.0)	9.9 ± 4.6 (9.5)	16.6 ± 4.0 (17.0)	< 0.001
Mean FFS-2 score (median)	5 ± 5 (3)	10 ± 6 (10)	16 ± 5 (17)	< 0.001

Abbreviations: FFS-1, Fear of Falls Scale balance-related motor ability subscore; FFS-2, Fear of Falls Scale fear subscore.

Notes: Group 1: Hoehn & Yahr stages 1–2; Group 2: Hoehn & Yahr stage 3; Group 3: Hoehn & Yahr stages 4–5. ^a^ Kruskal-Wallis Test.

#### *Patients with and without fear of falling (FFS-2)*

Among the patients with fear of falling, 90% presented worse FFS-2 scores ( *p* < 0.001, statistically significant) when compared to patients without fear of falling. The **Supplementary Material 3** (available at https://www.arquivosdeneuropsiquiatria.org/wp-content/uploads/2025/10/ANP-2025.0269-Supplementary-Material-3.docx ) presents the FFS-2 percentages for each question.

#### *Internal consistency of the FFS-Brazilian version*

The total internal consistency of the FFS-1 and FFS-2 and its individual FFS-1 and FFS-2 were evaluated by Cronbach's α scoring 0.96, 0.91, and 0.90 and by McDonald's ω scoring 0.96, 0.92, and 0.91, respectively. The values were considered sufficiently reliable. 16 Values of both the α and ω indices were close, which demonstrates that the instrument presented satisfactory reliability. 12 17

Assumptions for factorability were verified prior to dimensionality analysis. The KMO measure was 0.92, indicating sampling adequacy, and Bartlett's test of sphericity was significant (χ ^2^ = 1513.9; df = 190; *p* < 0.001), supporting factor analysis. An EFA was conducted using the RDWLS method with Promin rotation. Although the Kaiser criterion suggested retention of two factors (eigenvalues = 12.75 and 1.20), explaining approximately 69.7% of the total variance, the parallel analysis—considered more robust for ordinal data—indicated the presence of only one factor, which alone accounted for 65.45% of the total variance. Additionally, the two factors identified were highly correlated (r = 0.85), and most items loaded more strongly on the first factor, with only four items showing substantial loadings on the second factor.

Therefore, CFA were performed to examine the model structure further. The unidimensional model demonstrated adequate fit to the data, with χ ^2^ /df = 1.41, RMSEA = 0.054, CFI = 0.993, NNFI = 0.992, GFI = 0.952, and WRMR = 0.032, all within commonly accepted thresholds for model fit. In contrast, the bifactor model presented a poorer fit (χ ^2^ /df = 4.41; RMSEA = 0.157; CFI = 0.943; GFI = 0.706), indicating that the unidimensional structure provides a more appropriate representation of the scale.

#### *FFS-3*

Question 21 was descriptive, asking patients to identify any additional activities they feared or avoided due to fear of falling. Among the 140 respondents, 19 (13.5%) cited going up and down stairs, 10 (7.1%) walking on sidewalks, 7 (5.0%) playing football, 6 (4.2%) hiking, 5 (3.5%) navigating very steep hills, 4 (2.8%) driving, and 3 (2.1%) walking alone or crossing the street. Other activities were reported less frequently (2.7%).

#### *FFS-4*

Question 22 explored whether fear of falling was associated with the effect of PD medication, with 39% of patients reporting no such relation. The fear of falling increased for questions 23 and 24 during dyskinesia episodes and gait freezing.

## DISCUSSION

Fear of falling is a common complaint during PD assessments, especially in patients with greater disease severity. 1 A specific evaluation of this symptom enables healthcare professionals to personalize care and therapy for each patient. In this context, the FFS-Brazilian version fills this gap and helps identify specific situations of fear of falling associated with body balance.

Some instruments have already been used to assess the fear of falling in PD in different activities and daily life situations. 18 19 20 21 However, none of them present specific questions in relation to different motor abilities of static and dynamic balance. This FFS-Brazilian version is the first adaptation and validation to another language, in addition to the international design and validation study of the scale. The balanced sample across two regions likely minimized cultural bias.

Psychometric analyses of the FFS-Brazilian version provided robust evidence for the internal structure of the instrument. The initial exploration using EFA indicated mixed results regarding the number of latent factors, with the Kaiser criterion suggesting two dimensions, while parallel analysis supported a unidimensional structure. Considering the substantial correlation between factors and the factor loading pattern, a unidimensional interpretation was deemed plausible and theoretically parsimonious. The CFA results corroborated this interpretation, revealing superior fit indices for the one-factor solution compared to the bifactor model. These findings support conceptualization of the scale as measuring a single latent construct related to fear of falling. Given that sections 1 and 2 were conceptually designed to assess motor capacity and fear of falling respectively, the strong statistical and theoretical coherence found in the unidimensional model suggests these constructs may operate jointly in the experience of this fear in PD.

The FFS-Brazilian version showed good cross-cultural validity in the translation, back-translation and pretest processes. The high internal consistency in the total score and in FFS-1 and FFS-2 was also seen in the original validation study. 4 Internal consistency of both validations ensures high reliability. Furthermore, good content validity was verified, as the agreement rate judged by the expert committee was greater than 80%. 10

The majority of patients in our study reported “no difficulties” in FFS-1, and “no fear of falling” in FFS-2. These responses may be associated to a sample more concentrated in the HY stage 2, which, although bilaterally involved, does not present balance changes. These findings were also observed in the original validation study of the FFS, both regarding the number of patients who did not report fear of falling and the fact that the majority belonged to HY stage 2. 4 However, 45% of our patients reported a little or a lot of fear of falling, which corroborates with other studies that reported percentages of 36 to 58%. 22 23

In turn, the highest FFS-1 and FFS-2 scores were significantly related to PD severity in the correlations of MDS-UPDRS III, as well as with HY stage of PD. These findings were also evidenced in the literature and in the original scale of the study, correlating a greater fear of falling with the disease's more severe stages. 4 24

We found a higher score of the FFS-1 and HY in patients with fear of falling when compared to patients without a fear of falling. As PD progresses, difficulties in motor tasks related to balance increase, triggering greater fear of falling. 20 A qualitative study with 12 PD patients reported that fear worsened due to walking difficulties, hyperkinesia, rigidity, gait freezing, and balance impairments. 25 These findings are consistent with our validation and the original study, 4 where fear was associated with greater motor impairment. Patients with fear of falling also had significantly higher MDS-UPDRS scores, a finding mirrored in the original study. A retrospective study with 142 medical records found that as MDS-UPDRS worsened, particularly part III, avoidance behaviors became more evident. 26

In the FFS-Brazilian version, most patients with fear of falling reported “little fear” in FFS-2. Although few showed disabling fear, specific interventions are needed. Fear of falling exacerbates motor limitations and restricts daily activities. A study with 251 PD patients found that 70% of those with fear of falling avoided related activities, 20 similar to the 55% in our study. This behavior leads to reduced balance, increased weakness and lower physical conditioning, further worsening postural instability and fear, creating a vicious cycle. 27

Furthermore, 9 of the 10 questions in FFS-2 presented statistically significant differences when comparing responses from patients with and without fear of falling. These findings are important to demonstrate high sensitivity and reliability in determining this fear. The only nonsignificant question referred to the fear of falling in an elevator, which was not part of the routine of most interviewed patients.

This study has some limitations. Despite its multicenter design, generalization of the results should be approached cautiously, as most participants were in HY stage 2, typically without significant balance impairment or fear of falling. The exclusion of patients with cognitive impairment contributed to this sampling bias. Additionally, the sample was predominantly composed of individuals with over 7 years of education, which may have facilitated comprehension of the instrument.

Another point to consider is that a sample size calculation was not conducted. Nevertheless, the sample was based on the original FFS study (n = 95), which was sufficient for psychometric analysis. Future studies should include larger cohorts and more diverse samples, particularly patients in advanced PD stages, who present greater fear and risk of falling. Research is also needed to explore differences in fear of falling among PD phenotypes, specifically between postural instability and gait disorder and tremor-dominant subtypes. Although fear of falling is multifactorial, balance-focused interventions may help improve motor performance and reduce its effect on this population.

In conclusion, the current study provides a valid and reliable Brazilian version of the FFS for assessing fear of falling during daily activities, which involve motor skills related to postural balance in PD. Positive correlations were observed between fear of falling, the MDS-UPDRS, and disease severity. However, its application requires further investigation in different PD stages and in distinct sociocultural contexts.
